# Factors Affecting Nurse Retention Intention: With a Focus on Shift Nurses in South Korea

**DOI:** 10.3390/healthcare11081167

**Published:** 2023-04-18

**Authors:** Eun-Young Cho, Hwee Wee

**Affiliations:** 1 Department of Nursing, Wonkwang University Hospital, Iksan 54538, Republic of Korea; 2 Department of Nursing, Kunsan National University, Gunsan 54150, Republic of Korea

**Keywords:** shift nurses, stress response, work–life balance, grit, retention intention

## Abstract

This study aims to investigate the factors affecting shift nurses’ retention to solve the nurse shortage problem. The independent variables were general characteristics, stress response, work–life balance, and grit. The subjects for the study were 214 nurses working in three shifts at three general hospitals in Korea. Data were collected from 1 to 31 August 2022. We used structured tools such as the Nurses’ Retention Index, Stress Response Inventory, Work–Life Balance Scale, and Clinical Nurse’s Grit Scale. Data analysis was conducted using descriptive statistics, independent sample *t*-test, one-way variance analysis, Pearson correlation analysis, and hierarchical multiple regression analysis. Age, job satisfaction, and grit were factors that had a significant impact on retention intention. Grit had the greatest impact on retention intention. Additionally, retention intention increased in those aged from 30 to 40 years as compared with those under the age of 30. It is necessary to develop and implement a program that can improve grit to enhance the retention intention of shift nurses. Additionally, it is necessary to actively seek measures to reduce dissatisfaction with nursing jobs, increase satisfaction, and manage human resources considering the characteristics of age groups.

## 1. Introduction

The nursing profession makes up the largest section of healthcare employment and is a critical part of healthcare. However, around the world, the nursing turnover rate is high and there is a shortage of nurses [1]. In South Korea, the number of registered nurses was 436,340 [2] in 2020, but 51.67% were active nurses, meaning there were 225,462 working nurses. The largest percentage, 34.7%, worked for general hospitals. The nurse turnover rate was 19.7% [3]. The number of nurses per 1000 population increased from 2.34 in 2010 to 4.35 in 2020, but was very low as compared with the Organisation for Economic Cooperation and Development (OECD)’s average of 8.06 [4].

As the shortage of nurses exists regardless of the type of institution, the state, associations, and educational institutions should collaborate rather than transfer the problem of nurse employment and turnover to each medical institution. Recently, interest in retention intention, defined as the intention of a nurse to stop seeking another new job or to remain in the current nursing position [5], has increased. This can increase the retention of competent nurses by using retention, a positive concept, rather than turnover. Several studies identified factors associated with retention intention, such as demographic characteristics, job characteristics, environmental characteristics, and internal characteristics. A meta-analysis divided the factors influencing retention into external factors, such as nursing work and nursing environment, and internal factors, such as the attitude, nurse’s value, competence, and psychological resources [6]. However, as most of the surveyed factors focus on nursing jobs, it is necessary to confirm whether factors that consider nursing jobs and daily life at the same time affect retention. As suggested by previous studies [6], it is necessary to study psychological resources to increase retention. In particular, as shift work for nurses is not only a risk factor for stress and various diseases, but also an obstacle to social and family relationships [7], research on shift nurses is needed.

Stress has been viewed as a response, a stimulus, and a transaction. The way that individuals conceptualize stress determines the response, adaptation, or coping strategy, and the individual can experience it as eustress or distress [8]. In the stress response model, stress is defined in terms of response and, among the response patterns, distress is considered the pre-stage of dysfunction and disease [9]. Nurses experience intense stress because they must communicate with various people and cope with life-threatening situations [10]. As stress is mainly a problem and should be managed, investigating the relationship between distress and retention in the nurses’ overall life is needed.

For nurses to be happy and healthy, they should balance work and family, but nurses often experience a lack of balance between work and non-work life [11]. Work–life balance (WLB) refers to regulating and controlling life beyond work by appropriately distributing psychological and physical energy and time to leisure, family, and individual growth and development [12]. A staff nurse has little control over their work environment, but WLB can change this by taking action, such as self-reflection and recognizing the need for change [11]. As it has been reported that nurses’ WLB has reduced turnover intention [13] and affects job satisfaction to induce retention [14], it is worth investigating the relationship between WBL and retention.

Among the psychological resources, grit is a concept presented by Duckworth [15], defined as patience and passion for long-term goals, and is a way to make humans exercise their abilities. A gritty individual is characterized by striving hard for challenges while maintaining effort and interest for years despite failures and adversity. One can enhance grit with the help of parenting methods, mentors, teachers, managers, and supporters [16]. In recent years, grit has attracted attention as a psychological factor predicting an individual’s achievement in the workplace and influencing nurses’ turnover intention [17].

However, grit and turnover intention are positively correlated for nurses in rural areas. There are reports that grit is not an influencing factor in turnover intention [18], but an influencing factor only for comprehensive nursing care service unit nurses’ retention intention [19]. In Korea, when Duckworth’s [15] grit measurement tool was applied to nurses, internal reliability was low [17,19], and there was a limit to measuring the grit of nurses. Therefore, Park et al. [20] developed a grit measurement tool to be applied to clinical nurses. Therefore, it is necessary to continuously confirm the relationship between the clinical nurse’s grit and retention intention.

As the causes related to the shortage of nursing staff are various and concern international problems [1], continuous research is needed. However, to the best of our knowledge, no studies have confirmed whether general characteristics, stress response, WLB, and grit affect retention intention through hierarchical regression analysis. Therefore, this study intends to explore the direct effects of general characteristics, stress response, WLB, and psychological resource grit on retention intention as shift nurses’ personal factors. It can be used as elementary data to offer a plan to increase the retention of shift nurses by identifying the factors affecting retention.

This study aims to investigate the following: (1) the degree of variables; (2) the difference in retention intention according to general characteristics; (3) the correlation between stress response, WLB, grit, and retention intention; and (4) the effect of these variables on retention intention.

## 2. Materials and Methods

### 2.1. Research Design and Subject

This descriptive survey study aims to determine the effect of general characteristics, stress response, WLB, and grit on retention intention for nurses who work in shifts.

The participants were nurses who work in three shifts with more than three months of clinical experience at three general hospitals in Jeollabuk-do, South Korea. According to the Korean Labor Standards Act [21], three months is a legal standard for protecting employees from unfair dismissal of incumbent institutions. 

We used the G-power 3.1.9.4 program to calculate the number of samples. Based on previous studies [22,23], an effect size of 0.15, significance level of 0.05, power of 0.95, and 13 predictors (10 general characteristics and 3 independent variables) were calculated in the multiple regression analysis.

The minimum sample size was 189 people. We distributed a questionnaire to 218 people to provide for exclusions. We used 214 copies for the final analysis, excluding 4 unfaithfully made copies.

### 2.2. Research Tool

#### 2.2.1. Retention Intention

A retention intention tool was used [24] that translated the “Nurses’ Retention Index (NRI)” developed by Cowin [5] into Korean. There were six questions and two reverse questions in total. Using an eight-point Likert scale, scores ranged from 1 point for “not at all” to 8 points for “to a great extent”, and the score range was 6 to 48 points. The higher the score, the higher the retention intention. At the time of tool development [24], Cronbach’s α was 0.97, while it was 0.88 in the study using the modified tool and 0.90 in this study.

#### 2.2.2. Stress Response

For stress response, we used the Stress Response Inventory developed by Koh et al. [9] to measure stress, a factor affecting health. There 39 questions in total. On a five-point Likert scale, 0 represents “not at all” ranging to 4 points for “to a great extent”, and the score range was from 0 to 156. The higher the score, the higher the stress response. At the time of tool development [9], Cronbach’s α was 0.97 and, in this study, it was 0.97.

#### 2.2.3. Work–Life Balance (WLB)

For WLB, we used the Work–Life Balance Scale [12] developed for office workers, modified by Shin et al. [25] with appropriate phrases for nurses. There were 29 questions in total, including 8 questions for “harmony of work–family”, 8 for “harmony of work–leisure”, 9 for “harmony of work–growth”, and 4 for “general evaluation of life”. The seven-point Likert scale ranged from 0 points for “not at all” to 6 points for “to a great extent”, and the score range was 0 to 6. All of the questions were negative and treated as reverse questions. The higher the score, the higher the WLB level. In previous studies [25], Cronbach’s α for “harmony of work–family” was 0.79, “harmony of work–leisure” was 0.90, “harmony of work–growth” was 0.95, and “general evaluation of life” was 0.88. In this study, “harmony of work–family” was 0.81, “harmony of work–leisure” was 0.90, “harmony of work–growth” was 0.93, “general evaluation of life” was 0.90, and “work–life balance” was 0.96.

#### 2.2.4. Grit

For grit, we used the Clinical Nurses Grit Scale (CN-GRIT) developed by Park et al. [20], reflecting the culture and clinical nursing environment.

There were 14 questions—5 for “sustained persistence”, 5 for “consistency of interest as a nursing professional”, and 4 for “patient oriented intrinsic motivation”. On the four-point Likert scale, the score range was 14 to 56 points, from 1 point for “never” to 4 points for “always”. The higher the score, the higher the level of grit. At the time of tool development [20], Cronbach’s α was 0.91 and, in this study, it was 0.86.

#### 2.2.5. General Characteristics

There were 10 questions enquiring about sex, age, education, religion, marital status, subjective health status, clinical career, department, salary satisfaction, and job satisfaction.

### 2.3. Data Collection and Analysis

We conducted data collection from 1 to 31 August 2022. After obtaining permission to collect data from the head of the nursing department of the institution, a researcher or trained research assistant visited the participant in the ward and explained and distributed the questionnaire with written consent.

It took about 15 to 20 min to fill out the questionnaire and the completed questionnaire was collected in a sealed state. The study participants were offered small gifts. 

The collected data were analyzed using the IBM SPSS/WIN (version 23.0) statistical program. As a result of confirming the normality of each variable, the absolute value of skewness was 0.14~0.83, which was two or less, and the absolute value of kurtosis was 0.24~0.66, which was seven or less. The data for this study turned out to be normal. We used descriptive statistics for the subjects’ general characteristics and degree of variables. We conducted an independent *t*-test, one-way ANOVA, and a post hoc analysis with the Scheffé test to determine the difference in the retention intention according to the general characteristics. We used Pearson’s correlation coefficient for the correlation between variables and conducted hierarchical multiple regression analysis to identify the factors influencing retention intention.

### 2.4. Ethical Considerations

This study was approved by the Institutional Review Board of Kunsan National University (1040117-202206-HR-014-02) for research subjects’ rights and ethical considerations and was conducted with the permission of tool developers and modifiers to use the tool.

## 3. Results

### 3.1. General Characteristics of Subjects and Degree of Variables

The participants’ general characteristics and the average value of variables are shown in Table 1. Of the total 214 nurses, 186 (86.9%) were women and 28 (13.1%) were men. The average age was 29.86 ± 7.59 years. 

For the mean of each variable, retention intention was 31.52 ± 8.77 points, stress response was 40.50 ± 29.31 points, WLB was 2.84 ± 1.12 points, and grit was 39.57 ± 5.32 points.

### 3.2. Differences in Retention Intention According to the General Characteristics of the Subject

Regarding the general characteristics of the subject, retention intention differed according to the subject’s age (F = 3.22, *p* = 0.040), salary satisfaction (F = 9.87, *p* < 0.001), and job satisfaction (F = 36.43, *p* < 0.001). As a result of the post hoc analysis, salary satisfaction was 37.26 ± 7.64 points and the degree of retention intention was higher than that of other cases. For job satisfaction, retention intention was the highest, with 38.20 ± 6.59 points responding “satisfied”, followed by “so-so” with 31.15 ± 7.24 points and “dissatisfied” with 25.07 ± 9.35. According to the post-analysis results, there was no significant difference in age, as shown in Table 1.

### 3.3. Relations between the Subject’s Stress Response, WLB, Grit, and Retention Intention

The correlation between the subject’s stress response, WLB, grit, and retention intention is shown in Table 2. Retention intention has a significant negative correlation (r = −0.31, *p* < 0.001) with stress response. There was a significant positive correlation between WLB and retention intention (r = 0.28, *p* < 0.001), as well as between grit and retention intention (r = 0.59, *p* < 0.001).

### 3.4. Factors That Affect Retention Intention

We conducted a hierarchical multiple regression analysis to confirm the relative influence of factors affecting the retention intention of shift nurses, as shown in Table 3. Among the general characteristics of the subject, age, salary satisfaction, and job satisfaction, there were differences in retention intention, which were input by processing dummy variables. The three independent variables showed significant results in simple regression analysis and were placed under hierarchical multiple regression analysis. Before regression analysis, we identified multicollinearity between independent variables. The absolute value of the correlation coefficient of the independent variable was 0.28~0.59 and no variable with 0.80 or more appeared, confirming the independence of the variable. The tolerance limits were 0.59~0.93, which were 0.1 or higher, and the variance inflation factor (VIF) was 1.07~1.70, smaller than the reference value of 10; consequently, there was no problem with the multicollinearity of the independent variable. The Durbin–Watson index was 1.77 in Model 1 and 1.99 in Model 2, close to the reference value of 2.0. Consequently, there was no autocorrelation problem. We confirmed the linearity of the model, the normality of the error term, and equal variance.

The regression model of Model 1 was statistically significant (F = 14.27, *p* < 0.001). Among the general characteristics of the study subjects, job satisfaction satisfied (β = 0.33, *p* < 0.001) had the greatest impact, followed by job satisfaction dissatisfied (β = −0.29, *p* < 0.001), age 30 to under 40 (β = 0.17, *p* = 0.005), and age over 40 (β = 0.13, *p* = 0.040). The explanatory power of the four factors was 27.0%.

The regression model of Model 2 was statistically significant (F = 21.55, *p* < 0.001). Factors influencing the study subjects’ retention intention were grit (β = 0.45, *p* < 0.001), job satisfaction satisfied (β = 0.22, *p* = 0.001), job satisfaction dissatisfied (β = −0.22, *p* < 0.001), and age 30 to under 40 (β = 0.18, *p* = 0.001). The explanatory power of these four factors explaining the retention intention was 47.0% and grit has the greatest influence on the retention intention.

## 4. Discussion

The mean of the subjects’ retention intention was 31.52 out of 48 points, slightly higher than Kim’s [24] 27.88 points. It was slightly positive, converted to a rating of 5.25 out of 8 points, similar to 5.21 points in a previous study [23], which targeted general hospital nurses including full-time jobs. The mean of the stress response was 40.50 points, lower than the 68.5 points for general adults and 82.0 points for mentally disabled people reported in the study by Koh et al. [9]. The score converted to a rating was 1.04, which was at the level of “a little bit”. The mean of WLB was 2.84 points, at a slightly positive level, similar to the 2.99 points reported in a study of three-shift nurses [25]. The mean of grit was 39.57 points, slightly lower than the 42.94 points [26] surveyed online for nurses, including full-time positions at the general hospital. The score converted to a rating was 2.83 out of 4, a slightly positive level.

Retention intention according to the participants’ general characteristics differed according to age, salary satisfaction, and job satisfaction. Similar to this study, in the study of nurses working for more than three months in general hospitals [27], there were differences according to gender, age, marital status, education, perceived health status, type of work shift, current work unit, total clinical career, and job satisfaction. In the post hoc analysis, the study reported that those in their 40s and older had higher retention intention than those under 40. For job satisfaction, higher retention intention showed the satisfaction scales in the order of “satisfied”, “so-so”, and “dissatisfied”. Therefore, age and job satisfaction were commonly identified as general characteristics indicating the difference in retention intention. However, another previous study [27] included full-time nurses including the outpatient department, and the retention intention of three-shift workers was significantly lower than that of full-time workers, so the general characteristics of differences in retention intention for shift workers need to be confirmed through future studies.

The retention intention of shift nurses had a significant negative correlation with stress response and a significant positive correlation with WLB and grit. The correlation degree was moderate for grit (r = 0.59) and those for stress response (r = −0.31) and WLB (r = 0.28) were weak. Retention intention and grit are confirmed as positive correlations, but in the previous study [27], using Original Grit Scale (Grit-O) [15], the retention intention and grit had weak positive correlations (r = 0.25), making it difficult to accurately compare the tools and subjects used in this study. As grit is a psychological factor that predicts an individual’s achievement in the workplace [17], it is necessary to confirm the correlation between retention intention and grit through repeated studies considering future socio-cultural differences.

The first regression model identified the general characteristics affecting the retention intention of shift nurses. The items that influenced retention intention in the order relevance was “satisfied” for job satisfaction, “dissatisfied” for job satisfaction, age 30 to under 40, and age over 40. In the case of “satisfied” for job satisfaction compared with “so-so”, the retention intention increases and, in the case of “dissatisfied”, the retention intention decreases. If nurses feel dissatisfied with their job owing to shifts and overtime, they are likely to feel unhappy or unfulfilled during work [28], and nurses’ job satisfaction affects retention intention [6,14]. Therefore, it is necessary to improve the working environment so that nurses can be satisfied with their jobs and have positive thoughts during nursing work [6]. Many studies have reported that the age of nurses is related to retention intention [6]. In addition, as a result of this study, the influence on retention intention was higher for those over 30 years old than those under 30 years old. Therefore, it is necessary to investigate the variables related to the retention of nurses in each age group based on the age of 30 and to use the identified factors for managing nurses.

In the second model, stress response, WLB, and grit were added to the general characteristics. The factors influencing retention intention were grit, “satisfied” for job satisfaction, “dissatisfied” for job satisfaction, and age 30 to under 40. The explanatory power of these four factors explaining retention intention was 47.0%. The higher the grit, the higher the retention intention. This study showed that grit has the greatest direct influence on retention intention, supporting the result of another study using Grit-O [19] targeting nurses at the Comprehensive Nursing Care Service Unit. We confirmed that grit, a psychological factor predicting achievement, affects the retention intention of nurses performing shift work.

In nursing, grit has been studied in nurses since 2014 [29]. A conceptual analysis [30] was published in 2019 and clinical nurse grit measurement tools were developed in 2020 [20]. Grit predicts the achievement of individual nurses, and increasing grit can increase job performance [31]. Grit is an unrecognized feature of a changeable individual [16]; it increases long-term commitment to life and clarifies life purpose [17]. The management of grit levels for nurses is important in managing hospital human resources. Hwang and Nam [32] proposed an educational intervention strategy in cognitive, behavioral, and emotional areas that can improve grit, assuming that grit is malleable and teachable throughout life. It is necessary to develop and apply suitable interventions for nurses to improve grit and, in particular, the development and confirmation of effective programs for three-shift nurses [27] over full-time nurses being be prioritized for lower retention intention.

The results of this study can help prepare measures to maintain experienced nurses and capabilities of hospital organizations by increasing retention intention. Further, it was meaningful to use data during the COVID-19 pandemic to confirm that hospital shift nurses’ grit was a direct influence on their retention intention. In addition, it was found that stress response and WLB, which are factors that reflect daily life, do not affect retention intention. This may be owing to cultural influences, including excessive working hours in South Korea, which ranks fourth in the OECD [33], so studies targeting nurses in various countries are needed. Moreover, it suggests the need for a study to verify effectiveness by developing and applying a grit improvement program suitable for shift nurses.

## 5. Conclusions

As the shortage of nurse personnel is a global problem, nurses need to remain in their current nursing position. Nurses’ shift work can cause diseases and disabilities, and shift nurses’ retention intention is lower than that of full-time workers. Therefore, we investigated the influencing factors for shift nurses’ retention intention. The retention intention, WLB, and grit of shift nurses were slightly positive, and the stress response was at a level of “a little bit”, slightly lower than that of ordinary adults. Retention intention differed according to age, salary satisfaction, and job satisfaction. Retention intention had a negative correlation with WLB and grit. Among them, age, job satisfaction, and grit are factors that have a significant effect on retention intention, but grit has the greatest effect on retention intention. The results of this study can be used as evidence for ways to increase nurses’ retention intention and maintain their employment.

## Figures and Tables

**Table 1:** General characteristics of subjects, degree of variables, and differences in retention intention according to general characteristics (N = 214).

Variables	Categories	*n* (%)	Mean ± SD	F/t (*p*)	Scheffé
Sex	Female	186 (86.9)	31.72 ± 8.66	0.84 (0.400)	
	Male	28 (13.1)	30.21 ± 9.54		
Age (years)	<30	136 (63.6)	30.53 ± 9.23	3.22 (0.042)	
	30~<40	52 (24.3)	32.37 ± 7.68		
	≥40	26 (12.1)	35.00 ± 7.45		
	Mean ± SD	29.86 ± 7.59			
Education level	College	55 (25.7)	31.16 ± 8.36	2.32 (0.101)	
	University	140 (65.4)	31.10 ± 8.87		
	≥Graduate school	19 (8.9)	35.63 ± 8.60		
Religion	Have not	149 (69.6)	30.85 ± 8.77	−1.69 (0.093)	
	Have	65 (30.4)	33.05 ± 8.66		
Marital status	Unmarried	154 (72.0)	30.96 ± 8.84	−1.49 (0.137)	
	Married	60 (28.0)	32.95 ± 8.51		
Subjective health status	Good	50 (23.4)	33.68 ± 9.07	2.69 (0.070)	
	Moderate	129 (60.2)	31.27 ± 8.69		
	Bad	35 (16.4)	29.34 ± 8.20		
Clinical career (months)	<12	45 (21.0)	33.78 ± 8.31	2.48 (0.062)	
	12~<60	92 (43.0)	29.95 ± 9.10		
	60~<120	36 (16.8)	31.00 ± 9.01		
	≥120	41 (19.2)	33.02 ± 7.78		
	Mean ± SD	72.27 ± 87.69			
Department	General ward	43 (20.1)	31.86 ± 8.33	0.89 (0.448)	
	Intensive care unit	86 (40.2)	32.34 ± 8.65		
	Comprehensive nursing care service unit	55 (25.7)	29.91 ± 9.11		
	Emergency room	30 (14.0)	31.63 ± 9.14		
Salary satisfaction	Satisfied	27 (12.6)	37.26 ± 7.64	9.87 (<0.001)	b,c < a
	So-so	93 (43.5)	32.13 ± 8.31		
	dissatisfied	94 (43.9)	29.27 ± 8.75		
Job satisfaction	Satisfied	51 (23.8)	38.20 ± 6.59	36.43 (<0.001)	c < b < a
	So-so	117 (54.7)	31.15 ± 7.24		
	dissatisfied	46 (21.5)	25.07 ± 9.35		
Retension intention		31.52 ± 8.77			
Stress response		40.50 ± 29.31			
Work–life balance		2.84 ± 1.12			
Grit		39.57 ± 5.32			

**Table 2:** Correlation between retention intention and variables.

	Stress Response	Work–Life Balance	Grit
	r (*p*)		
Retention intention	−0.31 (<0.001)	0.28 (<0.001)	0.59 (<0.001)

**Table 3:** Factors affecting retention intention.

Variables		Model 1					Model 2				
		B	SE	β	t	*p*	B	SE	β	t	*p*
(constant)		30.33	0.89		33.95	<0.001	−0.03	4.17		−0.01	0.994
Age	30~<40 years *	3.48	1.24	0.17	2.81	0.005	3.76	1.08	0.18	3.48	0.001
	≥40 years *	3.37	1.63	0.13	2.07	0.040	1.82	1.46	0.07	1.25	0.215
Salary satisfaction	Satisfied *	0.29	1.91	0.01	0.15	0.880	−0.24	1.65	−0.01	−0.14	0.887
	Dissatisfied *	−0.88	1.19	−0.05	−0.74	0.463	−0.24	1.03	−0.01	−0.23	0.820
Job satisfaction	Satisfied *	6.73	1.51	0.33	4.47	<0.001	4.56	1.34	0.22	3.40	0.001
	Dissatisfied *	−6.12	1.41	−0.29	−4.36	<0.001	−4.73	1.27	−0.22	−3.71	<0.001
Stress response							−0.01	0.02	−0.01	−0.21	0.836
Work–life balance							0.02	0.02	0.07	1.12	0.265
Grit							0.73	0.09	0.45	7.97	<0.001
F (*p*)		14.27 (<0.001)					21.55 (<0.001)				
R^2^		0.29					0.49				
Adj R^2^		0.27					0.47				

* dummy variables; reference = age < 30 years, salary satisfaction so-so, job satisfaction so-so.

## Data Availability

Data are available upon substantiated request from the corresponding author.
